# Cyclodextrins as Drug Release Modulators and Toxic Compound Removal Agents

**DOI:** 10.3390/biom13071056

**Published:** 2023-06-29

**Authors:** Ana C. F. Ribeiro, Miguel A. Esteso

**Affiliations:** 1 CQC-IMS, Department of Chemistry, University of Coimbra, Rua Larga, 3004-535 Coimbra, Portugal; 2 Faculty of Health Sciences, Catholic University of Ávila, Calle Los Canteros s/n, 05005 Ávila, Spain

This Special Issue shows new strategies for the controlled release of drugs using cyclodextrins as carriers. Such advanced studies on model pharmaceutical formulations are useful since they allow the development of guidelines for the rational design of controlled-release systems.

Cyclodextrins (CDs), a family of cyclic oligosaccharides, are among the most widely used essential carriers. The basis for this popularity lies in the ability of these compounds to solubilize poorly soluble drugs, resulting in striking increases in their water solubilities and diffusion rates. In fact, due to their structure, they have a high capacity to form complexes with a large number of molecules (or ions). Therefore, they can be used as excipients for a large number of pharmacological preparations or as agents for eliminating unwanted ionic species in some media.

The first study [1] highlighted the relevant role of CDs in a particular drug delivery system targeting NPC or other diseases related to cholesterol imbalances in the brain. Moreover, these authors demonstrated that SPIONs (superparamagnetic iron oxide nanoparticles) appended to CDs provide a controlled way of simultaneously incorporating multiple functions that could benefit this kind of drug delivery system.

The next two studies [2,3] were focused on the possible aggregation between macrocycles (i.e., 2-hydroxypropyl-CD and β-CD) and other species (i.e., iodine and vanadium ions) and the loading efficiency of these components. For example, the study [3] revealed relevant findings from the analysis of the behavior of the diffusion of systems containing vanadium ions in the absence and presence of cyclodextrins. Namely, β-CD is the pharmacological compound capable of interacting with higher concentrations of vanadium ions in the oral cavity and those resulting from the tribocorrosion of the Ti-6Al-4V prosthetic.

In addition, in the last few decades, some work has been obtained in nanotechnological areas with medicinal applications [4,5], which may also involve macrocycles. For example, in reference [4], the authors have investigated the modulation of genes involved in cancer-associated canonical pathways induced by graphene engineered with cyclodextrins (GCD). These authors showed that the developed graphene-based drug delivery tool could offer an approach to the intracellular delivery of DOX and result in the maximization of its therapeutic effect and the limitation of its toxicity.

The characterization of multicomponent chemical systems is still poorly understood. However, it is very important as it helps us to better understand their structure and the behavior of the different drugs in media containing macrocycles in order to supply the technological and scientific communities with data on these important parameters and thus model them for practical applications.
